# Osteoimmunological impacts of micro/nanoplastics: systemic translocation, inflammatory responses, and bone remodeling disruption

**DOI:** 10.3389/fimmu.2026.1862512

**Published:** 2026-07-14

**Authors:** Jinzhu Fan, Jinmeng Li, Chao Hao, Yigeng Hui

**Affiliations:** Department of Orthopedic Microsurgery, Honghui Hospital, Xi’an Jiaotong University, Xi’an, China

**Keywords:** bone marrow niche, gut-immune-bone axis, immunotoxicity, leaky gut, micro/nanoplastics, osteoclastogenesis, osteoimmunology, osteoporosis

## Abstract

Ingested environmental micro- and nanoplastics (MNPs) may represent an emerging systemic health concern. Although toxicological research has mainly focused on the gastrointestinal tract, increasing evidence suggests that the highly vascularized bone marrow may also be a relevant site for MNP accumulation. This narrative review proposes a “gut-immune-bone” axis linking intestinal barrier disruption, systemic translocation, and potential deposition within the bone marrow niche. Current experimental evidence, together with limited human detection data, suggests that MNPs may disturb osteoimmunological homeostasis by impairing osteogenesis and promoting macrophage-associated osteoclastogenesis, thereby favoring bone remodeling imbalance. We summarize potential mechanisms, including oxidative stress, nuclear factor-κB (NF-κB) signaling, NOD-like receptor protein 3 (NLRP3) inflammasome activation, gut microbiota dysbiosis, endocrine disruption, and MNP-heavy metal co-exposure. We also discuss susceptible pediatric and geriatric populations and highlight the need to incorporate osteoimmunological endpoints into future MNP risk assessment.

## 1 Introduction

### 1.1 The global crisis of plastic pollution

Plastic pollution has emerged as a defining environmental challenge of the 21st century. Global plastic production has increased dramatically from approximately 2 million tonnes in 1950 to over 470 million tonnes in 2022, with projections indicating continued growth in the coming decades (1). To date, more than 8,000 million tonnes of plastics have been produced, of which nearly 80% have accumulated in landfills or the natural environment due to limited recycling and inadequate waste management (2). As a consequence, millions of tonnes of plastic waste enter aquatic ecosystems annually, making rivers and oceans the ultimate sinks for mismanaged plastics (3, 4).

Once released, plastics persist and progressively fragment into microplastics (MPs; generally defined as particles ranging from 1 μm to 5 mm) and, at smaller scales, nanoplastics (NPs; typically defined as particles < 1 μm), collectively referred to as micro- and nanoplastics (MNPs), which are now ubiquitous in freshwater, estuarine, and marine environments. Standardized global assessments reveal widespread contamination of surface waters, with particularly high concentrations in developing regions and major river systems, reflecting disparities in waste management infrastructure (5). Owing to their extreme persistence, both MPs and NPs are projected to accumulate to gigatonne levels in the environment by mid-century, even under scenarios of reduced production (6). This escalating and long-lasting pollution burden has intensified global concern and prompted the development of international, legally binding strategies aimed at controlling plastic pollution across its entire lifecycle (1).

### 1.2 Human exposure routes

Humans are exposed to plastic contaminants through multiple pathways, including ingestion, inhalation, and dermal contact (7). Owing to their distinct size regimes, MPs and NPs differ substantially in biological barrier penetration and intracellular trafficking, although they are often collectively discussed as MNPs in environmental and toxicological studies (8, 9). Despite these varied pathways, ingestion is widely regarded as the dominant exposure route due to the pervasive presence of plastic particles throughout the food chain (7). Among dietary sources, drinking water represents a major contributor to MNP intake. MPs have been detected in both tap and bottled water worldwide, with bottled water generally exhibiting higher particle concentrations, particularly in the micro- and nanoscale size ranges (10, 11). Based on typical consumption patterns, annual microplastic intake from drinking water alone may reach several hundred thousand particles for tap water consumers and several million particles for individuals relying exclusively on bottled water (12).

Aquatic food products constitute another critical ingestion pathway for human exposure to MNPs. Seafood, especially filter-feeding organisms such as bivalve mollusks, readily accumulates microplastics, facilitating their transfer to higher trophic levels, including humans (13, 14). Although estimated dietary intake from seafood varies across studies and analytical methods, consistent evidence confirms the presence of diverse plastic polymers in commercially available fish and shellfish (14, 15). Once ingested, MNPs interact with the gastrointestinal tract, where they may disrupt gut barrier function, immune homeostasis, and microbial composition, and have been detected in human feces and internal tissues (16, 17). Collectively, these findings underscore ingestion—particularly via drinking water and aquatic food products—as the primary route of human exposure to waterborne MNPs.

### 1.3 Bone as a neglected target organ

Current research on MNP toxicity has predominantly focused on the gastrointestinal tract, liver, lungs, brain, and reproductive organs, while bone tissue has remained largely overlooked as a potential target. Systematic analyses have confirmed the presence of MPs in multiple human tissues, including blood, placenta, lung, and gastrointestinal tract, leading toxicological assessments to primarily associate MNP exposure with digestive, respiratory, and reproductive health risks (18, 19). This organ-centric research paradigm has resulted in a substantial knowledge gap regarding skeletal exposure and toxicity, despite the systemic distribution potential of circulating MNPs.

Bone is a highly vascularized organ, and its unique bone marrow microvasculature—particularly the highly permeable sinusoidal vessels—renders it especially susceptible to blood-borne pollutants (20, 21). Recent studies have provided direct evidence of microplastic accumulation in human bone and bone marrow, identifying diverse polymer types within skeletal tissues (22–24). Experimental models further demonstrate that chronic MNP exposure disrupts bone microarchitecture, promotes osteoclastogenesis, and suppresses bone formation through oxidative stress and inflammatory signaling pathways (25–27). Analogous to fine particulate matter (PM2.5), which is now recognized as a systemic risk factor for osteoporosis and fractures (27), the detection of MPs in bone marrow raises serious concerns regarding their long-term impact on skeletal homeostasis and regenerative capacity, underscoring bone as a critical yet neglected target organ for MNP toxicity (25).

### 1.4 Aims of this review

This review bridges the critical knowledge gap between aquatic pollution and skeletal health by establishing the “gut-immune-bone” axis. We systematically trace the translocation trajectory of waterborne MNPs from gastrointestinal absorption to bioaccumulation within the bone marrow niche. Furthermore, we elucidate the underlying mechanisms of osteotoxicity—specifically focusing on oxidative stress, immunometabolic derailment, and gut microbiota dysbiosis—and evaluate their implications for future environmental risk assessment and public health management.

### 1.5 Literature search strategy

A comprehensive literature search was conducted in PubMed, Web of Science, and Scopus using keyword combinations such as “micro/nanoplastics,” “osteoimmunology,” “bone marrow niche,” and “gut microbiota.” The search spanned from database inception to May 2026, with a primary focus on recent toxicological advancements published between 2020 and 2026. Literature selection prioritized available human exposure and tissue-detection data, supplemented by *in vivo* and *in vitro* mechanistic studies. Because direct human causal evidence remains unavailable and many experimental studies use high-dose pristine particles, proposed pathways are interpreted according to their evidence strength.

## 2 Sources and characteristics of waterborne MNPs

MNPs have emerged as pervasive contaminants in aquatic environments, spanning freshwater, marine systems, and even drinking water supplies (28). Their widespread occurrence, diverse physicochemical characteristics, and strong interactions with coexisting pollutants collectively determine their environmental behavior and biological impacts (29). Understanding both the distribution patterns and intrinsic properties of waterborne MNPs is therefore essential for elucidating their transport, bioavailability, and potential health risks.

### 2.1 Occurrence in aquatic environments

MNPs are ubiquitous in aquatic environments, occurring across freshwater and marine systems with abundances spanning several orders of magnitude. In freshwater environments, including rivers and lakes, reported microplastic concentrations range from trace levels to over 10^5^ items/m³, with polyethylene (PE), polypropylene (PP), and polyethylene terephthalate (PET) as dominant polymers, and fibers and fragments as the prevailing morphologies (30, 31). Spatial variability is strongly influenced by population density, wastewater discharge, and atmospheric deposition, highlighting the role of human activity in shaping freshwater contamination patterns (31).

In marine environments, MNPs are widely distributed throughout the water column, sediments, and biota. Both MPs and NPs have been detected from surface waters to deep-sea ecosystems, with evidence suggesting that NPs may represent a substantial fraction of marine plastic pollution by mass (32, 33). Marine organisms across trophic levels are contaminated with MNPs, facilitating trophic transfer and bioaccumulation within marine food webs (34).

Drinking water represents a direct interface between aquatic contamination and human exposure. MPs are detected in the majority of tap water samples worldwide, with reported concentrations varying widely depending on analytical methods and particle size thresholds, and particles smaller than 50 μm predominating (10, 35). Bottled water generally contains higher concentrations of MNPs than tap water, particularly in the sub-micrometer to micrometer size range, with PE, PET, and PP as the most frequently identified polymers (36, 37). Together, these findings indicate that both natural waters and drinking water systems constitute significant reservoirs of MNPs and major sources of human exposure (**Table 1**).

**Table 1:** Global occurrence and primary dietary exposure routes of waterborne MNPs.

Environmental source/pathway	Key characteristics & dominant polymers	Human exposure context & accumulation burden	Ref
Freshwater Systems (Rivers & Lakes)	Concentrations range from trace levels to over 10^5^ items/m³. Dominant polymers include PE, PP, and PET, primarily as fibers and fragments.	Spatial variability is heavily driven by human population density, wastewater discharge, and atmospheric deposition.	(30, 31)
Marine Environments	MNPs are distributed from surface waters to deep-sea ecosystems. NPs represent a substantial fraction of marine pollution by mass.	Contamination across multiple trophic levels facilitates direct MNP bioaccumulation within marine food webs.	(32–34)
Tap Water	Particles smaller than 50 μm predominate in global tap water systems.	Annual intake for tap water consumers can reach several hundred thousand plastic particles.	(10, 12, 35)
Bottled Water	Exhibits higher MNP concentrations than tap water, particularly in the sub-micrometer range. Dominant polymers are PE, PET, and PP.	Exclusive reliance on bottled water can drive human consumption to several million particles per year.	(12, 36, 37)
Aquatic Foods (Seafood)	Filter-feeding organisms, such as bivalve mollusks, rapidly accumulate diverse microplastic polymers.	Acts as a critical dietary conduit, facilitating the trophic transfer of MNPs directly to humans.	(13–15)

### 2.2 Key physicochemical properties

#### 2.2.1 Size-dependent barrier penetration and bioavailability

**Figure 1:**
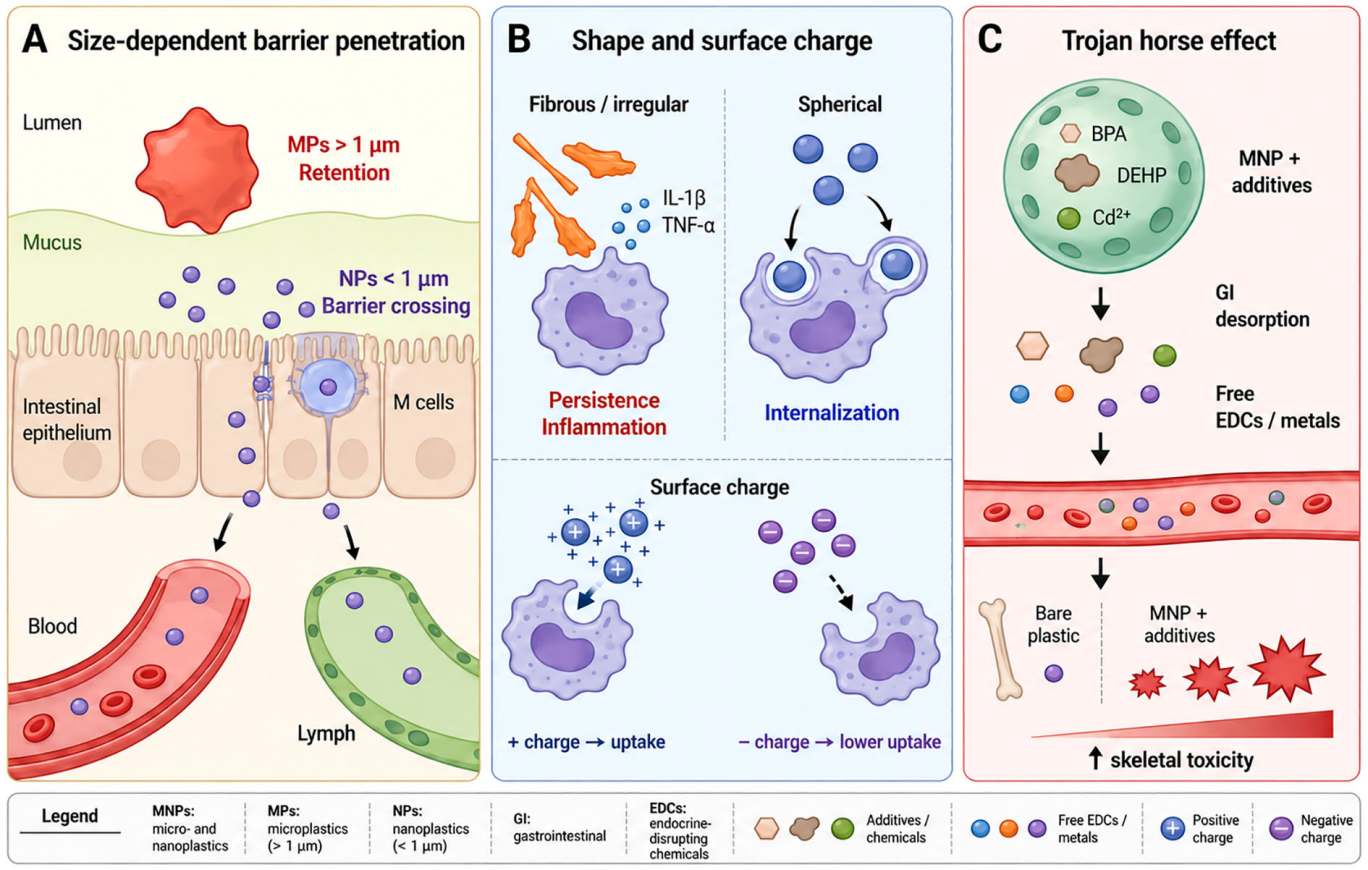
Physicochemical properties driving the bioavailability and targeted toxicity of MNPs. **(A)** Size-dependent barrier penetration: Larger MPs (> 1 μm) are predominantly retained or eliminated in the gastrointestinal tract, though a fraction accesses the lymphatic system via active microfold cell (M cell)-mediated transcytosis. Conversely, highly penetrative NPs (< 1 μm) readily cross the intestinal epithelium via paracellular routes and endocytosis to directly enter systemic circulation. **(B)** Morphological and electrostatic influences: Fibrous or irregularly shaped MNPs induce prolonged, frustrated phagocytosis and robust inflammatory responses, including the release of interleukin-1β (IL-1β) and tumor necrosis factor-α (TNF-α), while spherical particles are efficiently internalized. Additionally, positively charged particles (ζ-potential > 0 mV) exhibit enhanced cellular adhesion and uptake compared to highly negatively charged particles. **(C)** The “Trojan horse” effect: MNPs act as contaminant vectors, carrying endocrine-disrupting chemicals (EDCs), such as bisphenol A (BPA) and di(2-ethylhexyl) phthalate (DEHP), as well as heavy metals such as cadmium ions (Cd^2+^). Upon entering the acidic gastrointestinal environment, these non-covalently bound contaminants desorb, significantly increasing their bioaccessibility and exacerbating downstream systemic and skeletal toxicity compared to bare plastics.

The physicochemical properties of waterborne MNPs—specifically their size, shape, surface charge, and associated additives—are primary determinants of their bioavailability and systemic toxicity (**Figure 1**). Compared with MPs (> 1 μm), NPs (< 1 μm) more readily penetrate biological barriers, including the intestinal epithelium, blood–brain barrier, and placental barrier, owing to their small dimensions and high surface area-to-volume ratio (38–40). Experimental studies consistently demonstrate that NPs exhibit higher cellular uptake, enhanced translocation across epithelial layers, and broader tissue distribution, whereas larger MPs are more likely to be retained within the gastrointestinal tract or eliminated (38, 41).

#### 2.2.2 Shape and surface charge dynamics in cellular interactions

Particle shape and surface charge critically modulate interactions between MNPs and biological systems. Fibrous particles tend to persist longer in tissues and are associated with stronger inflammatory responses, while spherical particles are more efficiently internalized via endocytic pathways (42, 43). Surface charge, commonly characterized by ζ-potential, strongly influences particle–cell adhesion and uptake, with positively charged or weakly negative particles generally showing enhanced cellular internalization compared to highly negatively charged particles (44–46). Environmental aging and surface modification can further alter these properties, thereby affecting aggregation behavior and biological interactions. Crucially, it must be acknowledged that the majority of current toxicological data heavily rely on pristine, spherical polystyrene (PS) beads, which fail to fully recapitulate real-world exposures. In contrast to these pristine laboratory models, environmentally aged (weathered) MPs and NPs exhibit significantly altered surface charges, increased surface roughness, and higher porosity driven by ultraviolet photo-oxidation and mechanical degradation. These weathering-induced structural changes drastically amplify their capacity to adsorb and co-deliver secondary environmental pollutants via a “Trojan horse” effect (47), ultimately exacerbating systemic osteoimmunological toxicity.

#### 2.2.3 Chemical additives and the “trojan horse” effect

Beyond their intrinsic properties, MNPs can act as vectors for chemical additives and environmental contaminants, a phenomenon known as the “Trojan horse effect.” MNPs readily adsorb endocrine-disrupting chemicals (EDCs) such as bisphenol A (BPA) and phthalates, which may subsequently desorb in the gastrointestinal tract, increasing their bioaccessibility and systemic exposure (48). Experimental evidence indicates that MNPs carrying these additives can exacerbate cytotoxicity, oxidative stress, and genotoxic effects compared to exposure to either plastics or chemicals alone, with adsorption efficiency governed by polymer type, surface chemistry, and environmental conditions (49, 50).

## 3 The journey: bioaccumulation and translocation

Following environmental exposure, MNPs undergo complex biological transport processes that govern their internal distribution and accumulation. Their journey from the gastrointestinal tract to distant organs involves barrier penetration, systemic circulation, and tissue-specific deposition. Elucidating these pathways is essential for understanding how MNPs reach target sites such as bone and exert potential toxic effects.

### 3.1 Crossing the gut barrier

**Figure 2:**
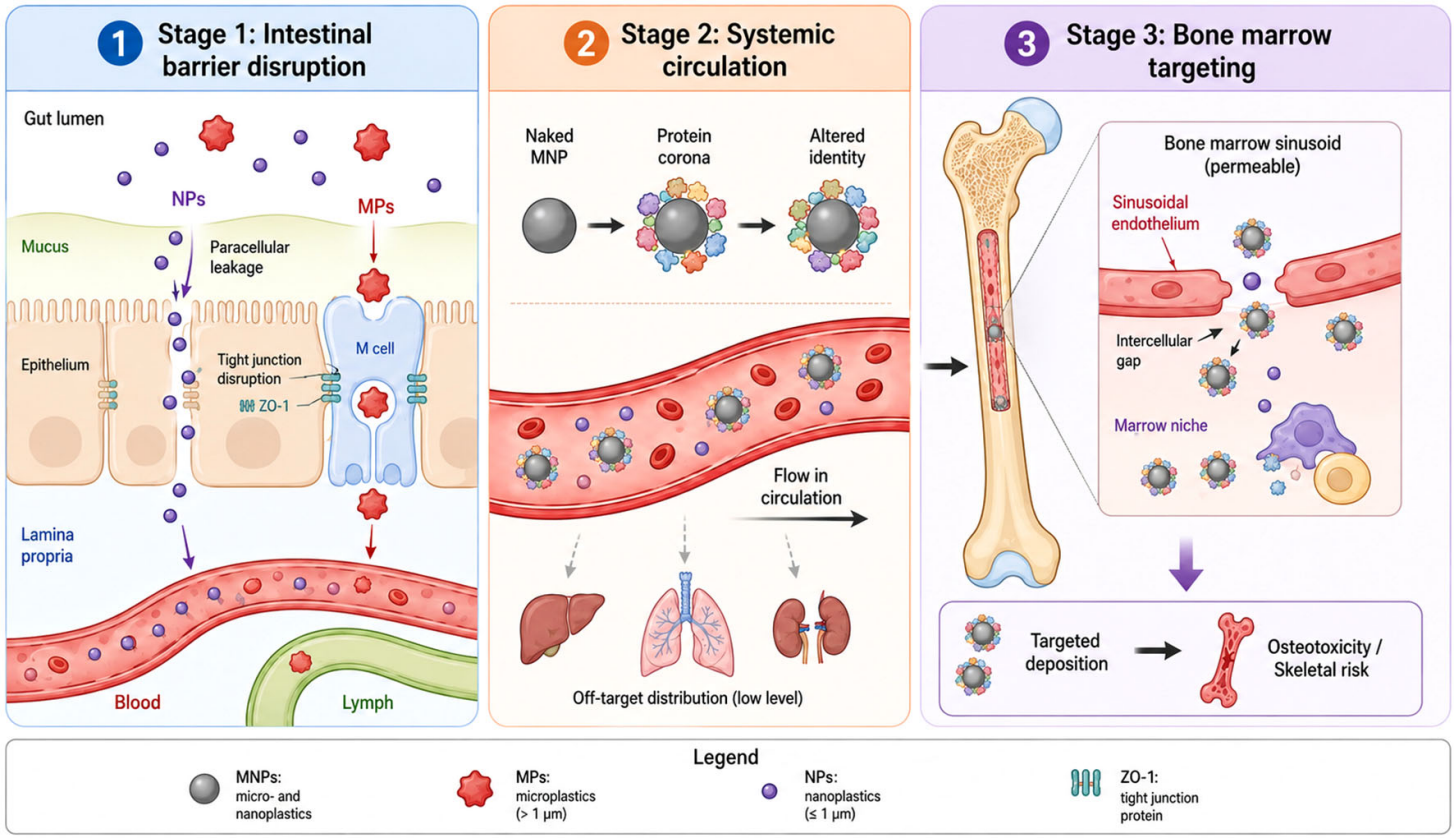
The three-stage anatomical translocation pathway of MNPs from the gastrointestinal tract to the bone marrow niche. (Stage 1) Intestinal barrier disruption and translocation: Plastic particles breach the gut epithelium via size-dependent routes: larger MPs undergo active M cell-mediated transcytosis, while NPs (< 1 μm) exploit paracellular leakage caused by the targeted downregulation of tight junction proteins (e.g., ZO-1, occludin). (Stage 2) Systemic circulation and protein corona formation: Upon entering the bloodstream, circulating MNPs rapidly adsorb plasma proteins (e.g., albumin, apolipoproteins, complement proteins), forming a “protein corona” that alters their biological identity and immune recognition. (Stage 3) Targeted accumulation in the bone marrow niche: Bone marrow sinusoidal vessels contain intercellular gaps reported up to approximately 61 nm, which may facilitate the extravasation of ultrasmall NPs or selected nanoscale particles. However, larger NPs and MPs are more likely to depend on endothelial uptake, protein-corona-mediated recognition, or monocyte/macrophage-assisted transport before accumulating in the skeletal microenvironment.

The systemic translocation of MNPs from the gastrointestinal tract to the skeletal microenvironment follows a distinct three-stage anatomical pathway (**Figure 2**). Initially, plastic particles cross the intestinal barrier via highly size-dependent routes: NPs readily enter systemic circulation through paracellular leakage across disrupted tight junctions, whereas larger MPs rely predominantly on active M cell-mediated transcytosis to access underlying lymphoid tissues (51). Due to their small size, NPs are capable of penetrating the intestinal mucus layer and interacting directly with the epithelial surface, where they are internalized by enterocytes via passive diffusion and active endocytic pathways, including clathrin-mediated and fast endophilin-mediated endocytosis (51, 52). In addition, M cells located in Peyer’s patches, which are specialized for luminal antigen sampling, facilitate the active transcytosis of larger MPs from the gut lumen directly into underlying lymphoid tissues (53, 54). This specific process provides a direct route for MPs to bypass epithelial defenses and access the underlying lymphatic system, complementing the systemic vascular distribution of NPs (54). Consistent evidence from *in vitro* and *in vivo* models indicates that NPs exhibit significantly higher uptake efficiency and translocation rates than MPs (54, 55).

Physical Injury to Intestinal Mucus and Tight Junctions. Within the gastrointestinal tract, plastic particles compromise gut barrier function through distinct, size-dependent physical mechanisms (56, 57). Large MPs act as mechanical irritants that abrade the protective mucin layer and severely disrupt the local microbiota niches (56), while NPs act as stealth penetrators, exploiting the resulting barrier permeability to directly assault epithelial tight junctions (57). Interaction with mucus components leads to adsorption of mucin proteins, increased particle aggregation, and altered surface charge, which collectively reduce mucus viscosity and thin the protective barrier, facilitating particle penetration (39). While NPs stimulate excessive mucus secretion and alter microbial colonization, MPs tend to deplete the mucus layer and promote intestinal dysbiosis (58). At the epithelial level, the direct assault by highly penetrative NPs downregulates tight junction proteins, including zonula occludens-1 (ZO-1), occludin, and tricellulin, resulting in increased paracellular permeability (59–61). These effects are mediated by inflammatory and oxidative stress–related signaling pathways, such as signal transducer and activator of transcription 1/6 (STAT1/6), extracellular signal-regulated kinase (ERK), and nuclear factor kappa B (NF-*κ*B), and are further amplified by cytokine release and microbiota imbalance (62–64). Gastrointestinal digestion further modifies nanoplastic surface properties through protein corona formation, enhancing their barrier-disruptive potential and cellular uptake (62).

### 3.2 Circulation and deposition

Following intestinal barrier disruption, plastic particles disseminate systemically via strictly size-dependent vascular routes. Specifically, NPs readily enter the blood circulation through paracellular leakage, whereas larger MPs predominantly access the lymphatic system following M cell-mediated transcytosis. *In vitro* tri-culture models and animal studies demonstrate that NPs are absorbed through passive diffusion and active endocytic pathways, including clathrin-mediated and fast endophilin-mediated endocytosis, as well as transcytosis by M cells in Peyer’s patches, which specialize in luminal antigen sampling and facilitate particle transport into underlying lymphoid tissues (51). The synergistic disruption of the mucus layer by MPs and the degradation of tight junction proteins (e.g., ZO-1, occludin, tricellulin) by NPs increase overall gut permeability, further promoting paracellular passage into systemic circulation (60, 65). Once in the bloodstream, MNPs are distributed to distal organs, including bone marrow. Human studies have directly detected MPs in bone marrow samples, with high detection rates for PE, PS, and other polymers, mostly smaller than 100 μm (22), and animal models confirm oral exposure leads to accumulation in bone tissue and marrow, causing hematopoietic toxicity and disruption of marrow cell arrangement (66). *In vitro*, NPs are readily internalized by bone marrow-derived phagocytic cells, including macrophages and dendritic cells, and are localized within phagosomes and the cytosol (67).

### 3.3 Bone marrow accumulation

The semi-open circulatory system of bone marrow is characterized by highly permeable sinusoidal vessels with intercellular gaps reported up to approximately 61 nm (68). These vascular openings may facilitate the extravasation and retention of ultrasmall NPs or selected nanoscale particles with compatible hydrodynamic diameters. However, because NPs are broadly defined as particles < 1 μm, not all NPs can be assumed to passively cross these fenestrations. Larger NPs and MPs are more likely to rely on endothelial uptake, protein-corona-mediated endothelial or immune recognition, immune cell-mediated transport, or macrophage “hitchhiking” to access or persist within the marrow niche. This size-restricted interpretation better explains divergent skeletal enrichment pathways without overgeneralizing passive vascular extravasation (68, 69).

Crucially, once MNPs enter systemic circulation, their biological identity is reshaped by rapid protein corona formation (70). The corona composition governs biodistribution and skeletal uptake, with hydrophobic plastic surfaces enriching apolipoproteins, complement component 3 (C3), and immunoglobulins (71). For penetrative NPs, apolipoprotein-rich coronas promote receptor-mediated uptake through scavenger and low-density lipoprotein (LDL) receptors on endothelial and bone marrow cells, shifting internalization toward clathrin-dependent endocytosis (72). In contrast, larger MPs enriched with complement factors and immunoglobulins undergo opsonization and are engulfed by monocytes/macrophages via complement and fragment crystallizable receptor (Fc receptor), enabling “hitchhiking” entry into marrow (73). Thus, the protein corona acts as a molecular “zip code” directing size-dependent MNP accumulation in the bone marrow niche (73).

Human studies confirm MNP detection in bone-related tissues, whereas experimental studies associate MNP exposure with impaired hematopoietic stem and progenitor cell function, reduced cell viability, and altered metabolic activity (26, 74). Systematic reviews and detection studies further confirm the presence of MNPs in human bone, cartilage, and marrow, highlighting bone as a vulnerable yet often overlooked target for systemic microplastic toxicity (75).

## 4 Potential effects on bone remodeling: experimental evidence and mechanistic inference

**Figure 3:**
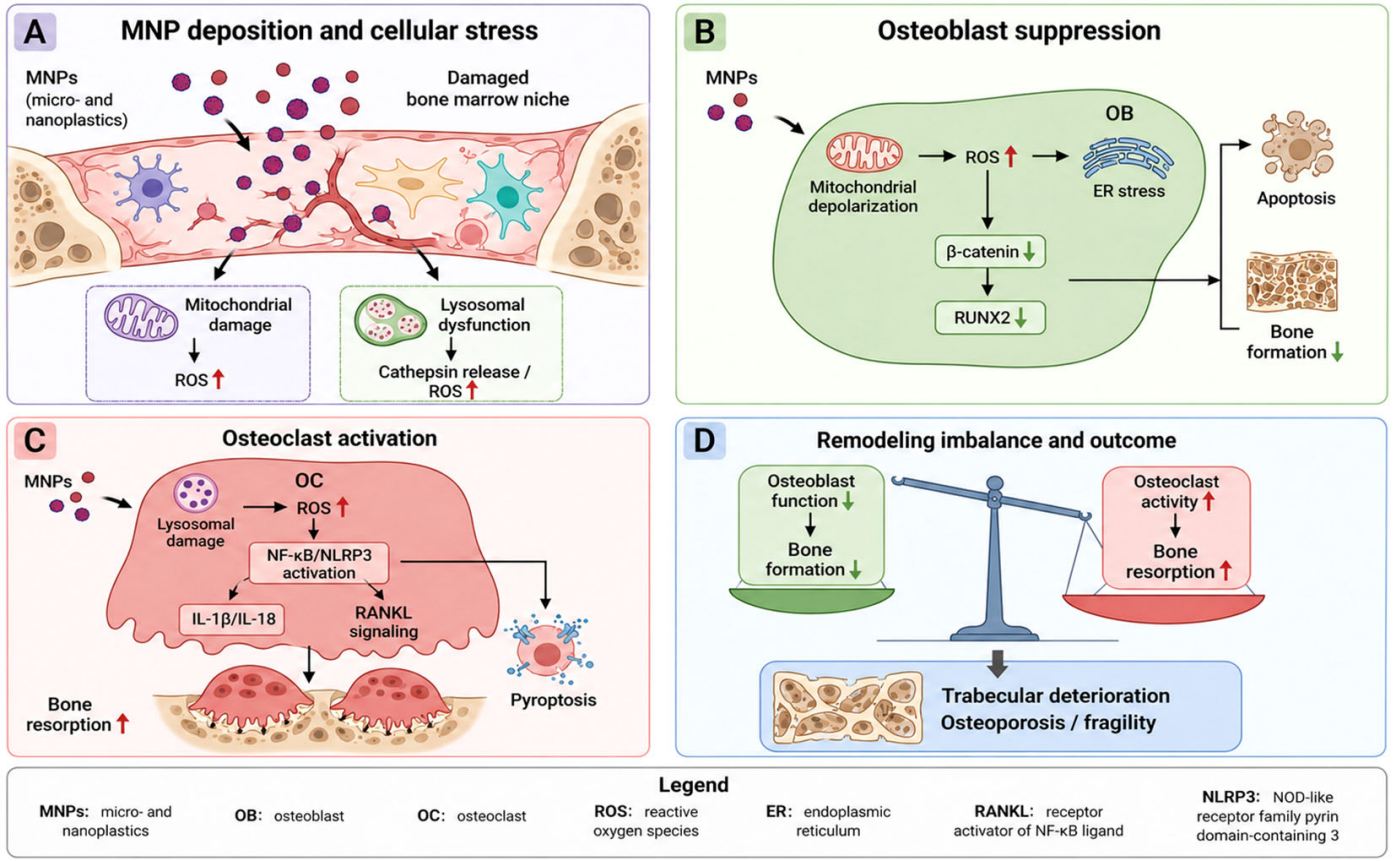
Potential mechanisms linking MNP exposure to bone remodeling imbalance within the bone marrow niche. **(A)** Size-dependent cellular stress: While MPs largely trigger extracellular or phagosomal inflammation, NPs (< 1 μm) may enter target cells and contribute to organelle stress, including mitochondrial depolarization and lysosomal dysfunction, thereby increasing reactive oxygen species (ROS) generation. **(B)** Inhibition of osteoblastogenesis: Internalized NPs may promote apoptosis and endoplasmic reticulum (ER) stress in osteoblasts. Simultaneously, MNP exposure is associated with suppression of the Wnt/β-catenin signaling pathway and downregulation of runt-related transcription factor 2 (RUNX2), which may impair osteoblast viability, matrix mineralization, and bone formation. **(C)** Activation of osteoclastogenesis: Internalization of MNPs by monocyte/macrophage lineages may activate the NF-κB and NLRP3 inflammasome pathways. This inflammatory cascade may increase RANKL expression, disrupt the RANKL/osteoprotegerin (OPG) ratio, and activate the receptor activator of nuclear factor-κB (RANK)-nuclear factor of activated T cells cytoplasmic 1 (NFATc1) signaling axis, thereby promoting osteoclast differentiation and bone resorption. **(D)** Skeletal homeostasis imbalance: The simultaneous suppression of bone-forming osteoblasts and activation of bone-resorbing osteoclasts may tilt the skeletal remodeling balance, potentially resulting in deteriorated bone microarchitecture and increased susceptibility to osteoporosis. Some immune links shown represent evidence-informed inferences from related osteoimmunology and wear-debris studies.

Upon accumulating within the bone marrow niche, MNPs may disrupt skeletal homeostasis by impairing osteogenesis and promoting osteoclastogenic signaling through interconnected molecular pathways (22, 25), as summarized in **Figure 3**. Experimental studies support oxidative stress and innate inflammatory responses, whereas the Th17/Treg–RANKL link remains an inference from broader osteoimmunology and wear-debris research (25, 76, 77).

### 4.1 Inhibition of osteogenesis

#### 4.1.1 Effects on osteoblast viability and mineralized nodule formation

MNPs inhibit osteogenesis by reducing osteoblast viability and impairing mineralized nodule formation. *In vitro* studies using murine pre-osteoblasts (MC3T3-E1) and human bone marrow stromal cells suggest that highly penetrative NPs can be internalized into the cytosol and nucleus, where they may induce oxidative stress, DNA damage, and caspase-3/7-associated apoptosis, thereby reducing osteoblast survival (23, 78, 79). Conversely, larger MPs primarily act at the cell surface or within phagosomes to promote cellular senescence and alter the differentiation potential of mesenchymal stromal cells, cumulatively compromising osteogenic function (25, 80). Experimental models also demonstrate that chronic exposure to PS NPs significantly decreases both the number and size of mineralized nodules, accompanied by reduced alkaline phosphatase activity and downregulation of osteogenic markers (23). Transcriptomic analyses of bone tissue from microplastic-exposed animals reveal broad suppression of osteogenesis-related genes and concurrent upregulation of inflammatory and metabolic stress pathways, including NF-κB, phosphoinositide 3-kinase/protein kinase B (PI3K-Akt), and hypoxia-inducible factor-1 (HIF-1) (81).

#### 4.1.2 Impairment of osteogenic signaling networks

MNPs interfere with key signaling pathways that govern osteoblast differentiation and bone formation. Notably, the Wnt/β-catenin pathway is suppressed *in vitro* and *in vivo* upon exposure to microplastics, as evidenced by decreased expression of Wnt target genes and β-catenin protein, resulting in impaired osteogenic commitment and reduced matrix deposition (26, 82). In addition, MPs modulate the RANKL/OPG axis, promoting osteoclastogenesis and enhancing bone resorption, thereby exacerbating bone loss (23). Collectively, these findings indicate that MNPs exert osteotoxic effects not only by compromising osteoblast viability and mineralization but also by perturbing critical osteogenic signaling networks, ultimately impairing bone regeneration and skeletal integrity.

### 4.2 Promotion of osteoclastogenesis

#### 4.2.1 Induction of monocyte/macrophage differentiation into osteoclasts

MNPs promote osteoclastogenesis by inducing differentiation of monocytes and macrophages into osteoclasts. Exposure to MNPs activates the NF-κB signaling pathway in bone marrow mesenchymal stem cells, accelerating cellular senescence and upregulating specific macrophage- and DC-derived cytokines (such as TNF-α and IL-6), which in turn stimulate RANKL overproduction and drive osteoclast differentiation from monocyte/macrophage precursors (25). *In vitro* studies show that MPs directly enhance osteoclastogenic commitment in RAW264.7 pre-osteoclasts, evidenced by increased expression of RANK, NFATc1, and other osteoclast-specific genes (23). NPs further potentiate osteoclastogenesis by inducing oxidative stress and apoptosis in pre-osteoclasts, sensitizing these cells to RANKL-mediated differentiation (26). MPs also modulate the bone marrow microenvironment, disrupting homeostasis and promoting recruitment and differentiation of monocyte/macrophage populations into osteoclasts (25). Chronic exposure impairs bone microstructure, reduces trabecular bone, and increases osteoclast activity *in vivo* (25), effects that are amplified by ROS generation and inflammatory signaling and are consistent across multiple polymer types including PE, PS, and polyvinyl chloride (83).

#### 4.2.2 Elevation of the RANKL/OPG ratio

A central mechanism driving MNP-induced osteoclastogenesis is the imbalance of the RANKL/osteoprotegerin (OPG) axis. Microplastic exposure increases RANKL secretion while suppressing OPG expression, resulting in a higher RANKL/OPG ratio and enhanced osteoclast formation (25, 84). This imbalance promotes bone resorption and exacerbates bone loss, as confirmed by transcriptomic analyses and functional validation in both animal and human bone tissue (23). The semi-open circulatory system of bone marrow facilitates direct interactions of MNPs with hematopoietic and stromal cells, further reinforcing osteoclastogenic signaling and matrix degradation (66). Collectively, these findings establish that MNPs drive osteoclast differentiation and bone resorption by activating monocyte/macrophage lineage commitment and increasing the RANKL/OPG ratio.

### 4.3 Potential adaptive immune involvement: Th17/Treg imbalance

Under physiological conditions, Treg cells limit osteoclastogenesis through IL-10, TGF-β, and CTLA-4 signaling (76). Direct MNP-specific evidence linking Th17/Treg dysregulation to skeletal loss remains limited. Based on MNP-associated IL-1β/IL-6 responses and analogous wear-debris biology, a Th17/Treg shift has been proposed but has not been directly verified in MNP-exposed bone marrow (77, 85–90). If present, IL-17 could increase RANKL expression and reinforce osteoclastogenesis, as established in inflammatory bone disease (90–93). Direct *in vivo* studies linking this axis to skeletal outcomes after environmentally relevant MNP exposure are still needed. It is important to note that while the impact of NPs on T cell polarization is becoming clearer, direct *in vivo* data tracking adaptive immune responses exclusively following macro- or microplastic (MP) exposure remains severely limited. Addressing this critical knowledge gap represents a vital frontier for future osteoimmunological research.

## 5 Mechanisms of action

**Figure 4:**
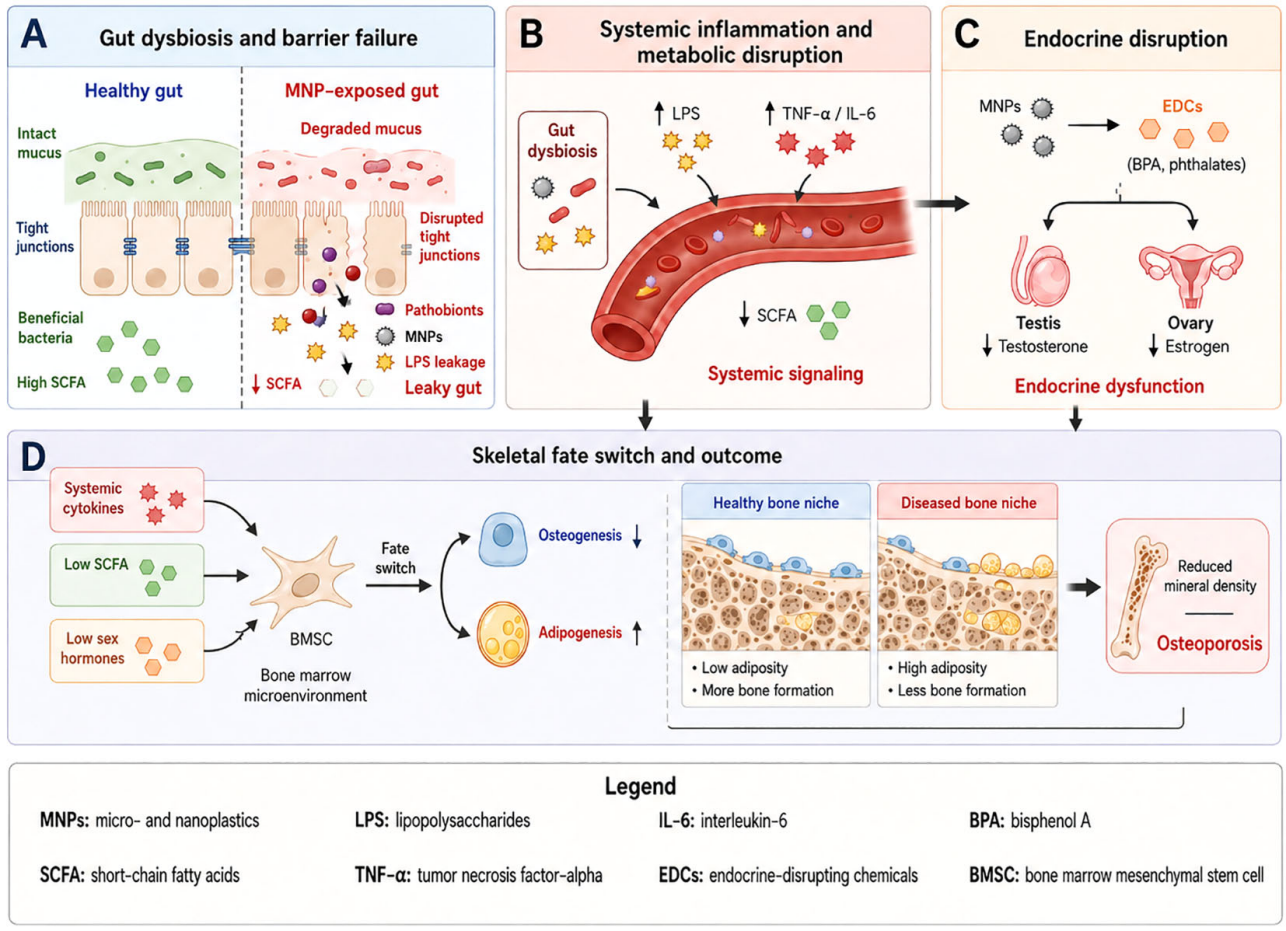
Plausible systemic pathways linking MNP exposure to osteotoxicity through the gut–immune–bone and endocrine axes. **(A)** MNP-induced gut microbiota dysbiosis and barrier failure: MNP exposure may impair the intestinal mucus layer and downregulates tight junction proteins (e.g., ZO-1, occludin), creating a “leaky gut” while severely depleting beneficial short-chain fatty acid (SCFA)-producing bacteria (e.g., Akkermansia). **(B)** Systemic inflammation and metabolic disruption: The compromised gut barrier allows for the systemic translocation of pathogenic lipopolysaccharides (LPS), potentially increasing systemic LPS and inflammatory cytokines (TNF-α, IL-6), alongside a critical reduction in osteoprotective SCFAs. **(C)** Endocrine disruption via MNP-released EDCs: MNPs act as vectors for EDCs like BPA and phthalates, which may disturb essential sex hormones (testosterone and 17β-estradiol) required for bone maintenance. **(D)** Convergent skeletal fate switch and disease outcomes: The synergistic systemic insults—high inflammation, diminished SCFAs, and suppressed sex hormones—may shift BMSC lineage commitment from osteogenesis toward adipogenesis. This pathological fate switch may contribute to a diseased skeletal microenvironment characterized by increased marrow adiposity, trabecular deterioration, and elevated osteoporosis susceptibility.

To avoid overinterpretation, we distinguish direct MNP-specific findings from pathways inferred from broader osteoimmunology, wear-debris, or non-skeletal MNP studies. Section 5.1 focuses on localized marrow injury, whereas **Figure 4** summarizes indirect systemic pathways.

### 5.1 Oxidative stress and inflammation

#### 5.1.1 Intrinsic mitochondrial stress and inflammation

MNP-induced bone toxicity appears to be closely associated with oxidative stress and inflammatory cascades that disrupt skeletal homeostasis. Owing to their subcellular dimensions, NPs may preferentially internalize into mitochondria and contribute to membrane depolarization and ROS overproduction, whereas larger MPs predominantly trigger extracellular or phagosomal ROS generation. Consequently, NP-associated mitochondrial dysfunction may promote cell-cycle arrest, DNA damage, and apoptosis across various bone cells (26, 80), while impairing adenosine triphosphate (ATP) production by disrupting vital inter-organelle communication (94).

Simultaneously, the extracellular and phagosomal ROS generated by larger MPs provoke a robust inflammatory response, upregulating pro-inflammatory cytokines, specifically TNF-α and IL-6, primarily through mitogen-activated protein kinase/extracellular signal-regulated kinase (MAPK/ERK) signaling pathways (95, 96), which alters bone morphogenetic cytokine expression (24). Mechanistically, MNP-induced osteoimmunotoxicity is governed by integrated signaling crosstalk rather than isolated pathway activation. Localized MNP stress may activate NF-κB as an upstream inflammatory node, which can transcriptionally prime the NOD-like receptor protein 3 (NLRP3) inflammasome to amplify pro-inflammatory cytokine release and contribute to RANKL/NFATc1-mediated osteoclastogenic signaling (23, 25). Concurrently, this inflammatory NF-κB/NLRP3 axis may inhibit downstream Wnt/β-catenin signaling in bone marrow stem cells, thereby suppressing osteoblastogenesis (26, 82). Together, these pathways form a pathological loop that may favor bone resorption while limiting bone formation, further exacerbated by cyclooxygenase-2 (COX-2)-mediated osteocyte senescence (80, 97).

#### 5.1.2 NLRP3 inflammasome activation and cellular pyroptosis

Experimental MNP models suggest inflammasome-related pyroptotic injury; direct confirmation in bone marrow macrophages *in vivo* remains limited. Larger MPs are engulfed via actin-dependent phagocytosis, frequently causing “frustrated phagocytosis” and sustained extracellular ROS production (98, 99). Conversely, smaller NPs are internalized via endocytic pathways, promoting lysosomal stress and enhanced NLRP3 activation (99, 100). Mechanistically, while amine-modified MNPs directly activate NLRP3, unmodified MNPs require damage-associated molecular patterns (DAMPs)-mediated priming through toll-like receptor 4 (TLR4), myeloid differentiation primary response 88 (MyD88), and NF-κB signaling (96, 101). Subsequent intracellular stressors—specifically lysosomal destabilization (cathepsin B leakage), mitochondrial ROS, and ion fluxes (K^+^ efflux/Ca^2+^ influx)—trigger NLRP3 to recruit apoptosis-associated speck-like protein containing a CARD (ASC) and activate caspase-1 (101). This promotes gasdermin D (GSDMD) cleavage, leading to cellular pore formation, pyroptotic lysis, and the release of IL-1β and interleukin-18 (IL-18) (101). Ultimately, this pyroptotic inflammatory niche may enhance RANKL-mediated osteoclastogenesis while impairing osteoblast mineralization (25, 102).

#### 5.1.3 Lipid peroxidation and cellular ferroptosis

Crucially, MNP-mediated “Trojan horse” delivery of heavy metals may induce ferroptosis, an iron-dependent regulated cell death driven by lipid peroxidation, within bone tissue (103). Acidic gastrointestinal and lysosomal microenvironments promote intracellular release of adsorbed metal ions, such as Cd^2+^ and lead ions (Pb^2+^), disrupting systemic and local iron homeostasis (104). Together with NP-associated mitochondrial depolarization, this intracellular metal burden may enhance Fenton chemistry, leading to increased hydroxyl radical and ROS generation (105). Concurrently, MNP–heavy metal co-exposure depletes glutathione (GSH) and suppresses the system Xc^−^/glutathione peroxidase 4 (GPX4) axis, weakening the major defense against lipid peroxidation (106). As GPX4 activity declines, skeletal cells fail to clear lipid peroxides (LPOs), causing membrane damage and ferroptotic death of BMSCs and osteoblasts (26). This process may impair the regenerative cell pool, thereby contributing to reduced bone mineral density and trabecular deterioration (107).

### 5.2 The “gut-immune-bone axis”

MNP exposure may disrupt the “gut-immune-bone axis” through synergistic microbiota dysbiosis and intestinal barrier dysfunction. Physically, larger MPs can degrade the protective mucus layer and reduce beneficial bacteria such as Akkermansia (108, 109), while highly penetrative NPs may trigger oxidative stress and compromise tight junction proteins, contributing to a “leaky gut” phenotype (110). This dual disruption may favor bone resorption via two converging pathways. First, the hyperpermeable barrier allows lipopolysaccharides (LPS) to translocate into systemic circulation, activating TLR4 signaling in immune cells and osteoclast precursors and thereby promoting osteoclastogenesis and trabecular bone loss (111, 112). Concurrently, depletion of short-chain fatty acid (SCFA)-producing bacteria, such as Bifidobacterium and Faecalibacterium, may reduce butyrate, acetate, and propionate levels (111, 113). Because SCFAs normally maintain bone homeostasis by supporting osteoblast differentiation through Wnt/insulin-like growth factor-1 (IGF-1) signaling and suppressing osteoclastogenesis through metabolic reprogramming, free fatty acid receptor 2 (FFAR2)/G protein-coupled receptor 43 (GPR43) activation, NF-κB inhibition, and Treg support (114–116), SCFA deprivation may synergize with LPS-driven endotoxemia and disrupted amino acid metabolism to shift bone remodeling toward net bone loss (117).

### 5.3 Endocrine disruption

MNPs may disrupt skeletal endocrine regulation both through particle-associated effects and by acting as vectors for EDCs such as bisphenols (BPA) and phthalates (118). By structurally mimicking hormones, these leached EDCs may reduce essential sex hormone levels, including testosterone and 17β-estradiol, via hypothalamic-pituitary-ovarian axis disruption (119, 120). Concurrently, they may inhibit osteogenic differentiation, as reflected by suppressed RUNX2 and alkaline phosphatase expression (121, 122). Beyond disrupting sex hormones, highly penetrative NPs specifically cross glandular endothelial barriers to induce thyroid follicular cell pyroptosis and alter parathyroid hormone synthesis. This deep-tissue penetration profoundly impairs the systemic calcium homeostasis strictly required for maintaining healthy bone metabolism (123, 124).

### 5.4 Age-related susceptibility: immunosenescence and inflammaging

Aging exacerbates MNP osteoimmunotoxicity through immunosenescence and inflammaging. Senescent BMSCs and hematopoietic stem cells (HSCs) accumulate in the aged marrow niche and release senescence-associated secretory phenotype (SASP) factors, including IL-6, IL-8, and TNF-α, creating chronic low-grade inflammation that impairs osteogenesis and favors adipogenesis (125, 126). Meanwhile, thymic involution, chronic antigen exposure, and Treg dysfunction shift the Th17/Treg balance toward a pro-osteoclastogenic state (127, 128). Upon MNP exposure, this pre-existing inflammatory baseline synergizes with frustrated phagocytosis, lysosomal damage, and mitochondrial dysfunction, amplifying IL-17 secretion, RANKL expression, and osteoclastogenesis (128, 129). Thus, SASP accumulation, Treg impairment, and myeloid bias may biologically prime the elderly skeletal niche for enhanced bone loss and increased susceptibility to senile osteoporosis following MNP exposure (128, 129).

## 6 Environmental risk assessment & public health implications

Translating experimental mechanisms into public health strategies requires a comprehensive evaluation of real-world exposure realities. The overall risk of MNP-induced osteotoxicity is heavily modulated by several amplifying factors, including human equivalent dose (HED) discrepancies, the heightened physiological vulnerabilities of pediatric and geriatric populations, and the synergistic toxicity of co-exposure with heavy metals.

### 6.1 Human equivalent dose conversion

A critical gap exists between the high MNP doses in animal studies and realistic human exposure, limiting translational validity (130). Humans ingest roughly 39,000–121,000 particles annually through food and inhalation, or 122–203 mg per day, heavily influenced by bottled water consumption (131). However, most *in vivo* models use commercially sourced MNPs at unrealistically high concentrations (132). Some low-dose or environmentally relevant models report gut dysbiosis, metabolic alterations, and low-grade inflammation; however, heterogeneity in particle characteristics, dose metrics, and exposure duration precludes reliable extrapolation to human skeletal risk (133, 134). Future research must prioritize standardized, long-term studies using realistic environmental MNP mixtures to enable accurate human health risk assessments.

### 6.2 Vulnerable populations

Children are exceptionally vulnerable to MNP toxicity due to higher intake-to-body-weight ratios, hand-to-mouth behaviors, and immature detoxification systems (135). During critical developmental windows, MNP exposure disrupts epiphyseal growth plate chondrocyte differentiation and induces endochondral ossification disorders via the protein kinase R-like endoplasmic reticulum kinase/eukaryotic initiation factor 2 alpha (PERK/eIF2α) endoplasmic reticulum stress pathway, leading to growth plate thinning and skeletal growth retardation (136). Conversely, elderly individuals face compounded risks due to age-related declines in bone remodeling (25). In the senescent niche, MNP exposure may interact with myeloid-biased inflammaging, enhance SASP-associated IL-6/TNF-α signaling and RANKL expression, and concurrently impair intestinal vitamin D receptor (VDR) signaling, thereby potentially increasing susceptibility to age-related bone loss and osteoporosis (137, 138).

### 6.3 Combined toxicity

MNPs act as highly efficient vectors for heavy metals like cadmium (Cd) and lead (Pb) through surface complexation and electrostatic interactions (139). Environmental aging and smaller particle sizes further enhance this toxic adsorption capacity (140). Crucially, MNPs exert a “Trojan horse” effect, where acidic gastrointestinal conditions trigger massive intracellular metal release (141, 142). This combined exposure severely compromises the intestinal barrier, amplifying systemic toxicity (56). Ultimately, co-exposure to MNPs and heavy metals may aggravate bone homeostasis imbalance and contribute to reduced bone mineral density and increased osteoporosis susceptibility through oxidative stress and ferroptosis (57, 143). A comprehensive summary of these critical risk amplification factors—including dosage disparities, uniquely susceptible life stages, and combined toxicological exposures—is provided in **Table 2**.

**Table 2:** Risk amplification factors and susceptible populations potentially associated with MNP-related osteotoxicity.

Risk factor/vulnerable population	Underlying pathological & toxicological mechanisms	Clinical & skeletal outcomes	Ref
Pediatric Population (Children)	Children possess immature detoxification systems, hand-to-mouth behaviors, and higher MNP intake-to-body-weight ratios.	MNP exposure may induce ER stress and disrupt growth plate chondrocyte differentiation, potentially impairing endochondral ossification and skeletal growth.	(135, 136, 138)
Geriatric Population (Elderly)	Aging naturally accelerates the decline of baseline bone remodeling. MNPs potentially bioaccumulate inside human vertebrae and limb bones.	Chronic exposure may further accentuate age-related bone mineral density loss and increase susceptibility to osteoporosis, although direct causal evidence in older humans remains limited.	(25, 137)
Combined Exposure (Heavy Metals)	MNPs may adsorb or complex heavy metals such as Cd and Pb, with gastrointestinal conditions promoting contaminant desorption and local bioaccessibility.	Synergistic co-exposure may aggravate intestinal barrier dysfunction and contribute to bone homeostasis imbalance through oxidative stress, inflammatory signaling, and ferroptosis-related pathways.	(56, 57, 139, 141–143)
HED Discrepancy	Estimated human MNP intake is approximately 122–203 mg per day, yet many in vivo studies rely on unrealistically high concentrations.	Low-dose or environmentally relevant exposure models have been associated with gut dysbiosis, lipid metabolic alterations, and low-grade inflammatory responses, although dose extrapolation to humans remains uncertain.	(131–134)

## 7 Management strategies and future perspectives

### 7.1 Advanced water treatment

Conventional wastewater treatment plants (WWTPs) can eliminate up to 99% of MPs but are less effective at capturing NPs, often transferring retained particles into agricultural sludge (131). Furthermore, mechanical shear forces within WWTPs may generate secondary NPs (131).

While membrane bioreactors and ultrafiltration achieve superior removal rates exceeding 99%, they remain hindered by severe membrane fouling and high costs (132, 133). Alternatively, coagulation-flocculation-sedimentation offers a cost-effective physical enhancement, while advanced oxidation processes (AOPs) show promise for active degradation (144, 145). To reduce the potential systemic translocation of NPs and subsequent activation of bone marrow inflammasome-related pathways, future water treatment frameworks should consider ultrafiltration thresholds (< 100 nm) and shift from merely relocating plastics toward more complete degradation or chemical mineralization.

### 7.2 Policy recommendations

Current environmental health risk assessments for emerging MNP pollutants predominantly focus on gastrointestinal, reproductive, and cardiovascular toxicities, while skeletal vulnerabilities remain insufficiently considered. Given the emerging evidence of MNP-associated osteotoxicity involving oxidative stress, endocrine disruption, and the gut-immune-bone axis, regulatory frameworks should consider incorporating skeletal endpoints into future assessment models. Candidate exploratory endpoints may include the RANKL/OPG ratio, Th17/Treg balance, systemic SASP cytokines, and BMD trajectories, but their MNP-specific predictive value requires validation. Establishing targeted skeletal risk assessment protocols may help better protect vulnerable populations and inform more comprehensive public health policies against plastic pollution.

### 7.3 Future research gaps

Despite advances, direct causal evidence linking human MNP exposure to clinical skeletal outcomes remains absent. Current models are often high-dose and pristine-particle based, limiting quantitative extrapolation to environmental exposure and clinical risk. Furthermore, assessments largely ignore critical confounders modulating bone metabolism: diet, sex, hormones, and baseline gut microbiota. To bridge these gaps, future paradigms must prioritize environmentally relevant low-dose exposure and real-world mixed pollution. Ultimately, longitudinal cohort studies rigorously controlling for these covariates are needed to determine whether MNP exposure is associated with BMD alterations and osteoporosis risk.

Finally, we acknowledge that our review primarily focuses on oral ingestion. Inhalation of airborne NPs represents a critical, yet under-addressed, pathway that bypasses the gastrointestinal barrier to facilitate direct systemic translocation. Future toxicological models must integrate this inhalation-driven influx to more accurately map the full spectrum of skeletal risks and the comprehensive human bone toxicokinetics required for evidence-based clinical guidelines.

## 8 Conclusion

Waterborne MNPs may represent an emerging environmental risk factor for skeletal metabolic health. Through the gut-immune-bone axis, ingested MNPs may disrupt intestinal barrier integrity, enter systemic circulation, and potentially reach or persist within the bone marrow niche. Current experimental and limited human evidence suggests that MNP exposure may impair skeletal homeostasis by suppressing osteogenesis, promoting osteoclastogenic signaling, and amplifying oxidative stress, immunometabolic dysregulation, gut microbiota dysbiosis, and endocrine disruption. Recognizing MNPs as potential skeletal risk modifiers may help refine future environmental risk assessment and guide public health strategies for vulnerable populations.

## References

[1] Landrigan PJ Dunlop S Treskova M Raps H Symeonides C Muncke J . The Lancet Countdown on health and plastics. Lancet (London England). (2025) 406:1044–62. doi: 10.1016/S0140-6736(25)01447-3 40769171

[2] Geyer R Jambeck JR Law KL . Production, use, and fate of all plastics ever made. Sci Adv. (2017) 3:e1700782. doi: 10.1126/sciadv.1700782 28776036 PMC5517107

[3] Borrelle SB Ringma J Law KL Monnahan CC Lebreton L McGivern A . Predicted growth in plastic waste exceeds efforts to mitigate plastic pollution. Sci (New York NY). (2020) 369:1515–8. doi: 10.1126/science.aba3656 32943526

[4] Lebreton LCM van der Zwet J Damsteeg J-W Slat B Andrady A Reisser J . River plastic emissions to the world’s oceans. Nat Commun. (2017) 8:15611. doi: 10.1038/ncomms15611 28589961 PMC5467230

[5] An X Yang Y Zhao X Zhu L Kang Y An L . Global measurement of surface water microplastics using a unified size threshold. Water Res. (2026) 288:124622. doi: 10.1016/j.watres.2025.124622 40986999

[6] Schwarz AE Lensen SMC Langeveld E Parker LA Urbanus JH . Plastics in the global environment assessed through material flow analysis, degradation and environmental transportation. Sci Total Environ. (2023) 875:162644. doi: 10.1016/j.scitotenv.2023.162644 36889399

[7] Wu P Lin S Cao G Wu J Jin H Wang C . Absorption, distribution, metabolism, excretion and toxicity of microplastics in the human body and health implications. J Hazard Mater. (2022) 437:129361. doi: 10.1016/j.jhazmat.2022.129361 35749897

[8] Zhou G Li J Chen Y Cui Y Wang Y Zheng L . Size-dependent toxicological effects of microplastics: A review. Ecotoxicology Environ Saf. (2026) 311:119831. doi: 10.1016/j.ecoenv.2026.119831 41653857

[9] Dong Y Pang M Li Y Li J Zhang C Sun G . Size-dependent toxicity of microplastics and nanoplastics: Insights from the Drosophila melanogaster model. Xenobiotica; fate foreign compounds Biol Syst. (2026) 56(5)414–423. doi: 10.1080/00498254.2026.2671851 42136038

[10] Sun T Teng Y Ji C Li F Shan X Wu H . Global prevalence of microplastics in tap water systems: Abundance, characteristics, drivers and knowledge gaps. Sci Total Environ. (2024) 929:172662. doi: 10.1016/j.scitotenv.2024.172662 38649043

[11] Li H Zhu L Ma M Wu H An L Yang Z . Occurrence of microplastics in commercially sold bottled water. Sci Total Environ. (2023) 867:161553. doi: 10.1016/j.scitotenv.2023.161553 36640894

[12] Danopoulos E Twiddy M Rotchell JM . Microplastic contamination of drinking water: A systematic review. PloS One. (2020) 15:e0236838. doi: 10.1371/journal.pone.0236838 32735575 PMC7394398

[13] Danopoulos E Jenner LC Twiddy M Rotchell JM . Microplastic contamination of seafood intended for human consumption: A systematic review and meta-analysis. Environ Health Perspect. (2020) 128:126002. doi: 10.1289/EHP7171 33355482 PMC7757379

[14] Cho Y Shim WJ Jang M Han GM Hong SH . Abundance and characteristics of microplastics in market bivalves from South Korea. Environ pollut (Barking Essex: 1987). (2019) 245:1107–16. doi: 10.1016/j.envpol.2018.11.091 30682745

[15] Ribeiro F Okoffo ED O’Brien JW Fraissinet-Tachet S O’Brien S Gallen M . Quantitative analysis of selected plastics in high-commercial-value Australian seafood by pyrolysis gas chromatography mass spectrometry. Environ Sci Technol. (2020) 54:9408–17. doi: 10.1021/acs.est.0c02337 32644808

[16] Hartmann C Lomako I Schachner C El Said E Abert J Satrapa V . Assessment of microplastics in human stool: A pilot study investigating the potential impact of diet-associated scenarios on oral microplastics exposure. Sci Total Environ. (2024) 951:175825. doi: 10.1016/j.scitotenv.2024.175825 39197786

[17] Özçifçi Z Basaran B Akçay HT . Microplastic contamination and risk assessment in table salts: Turkey. Food Chem toxicology: Int J published For Br Ind Biol Res Assoc. (2023) 175:113698. doi: 10.1016/j.fct.2023.113698 36889431

[18] Zhu L Kang Y Ma M Wu Z Zhang L Hu R . Tissue accumulation of microplastics and potential health risks in human. Sci Total Environ. (2024) 915:170004. doi: 10.1016/j.scitotenv.2024.170004 38220018

[19] Chartres N Cooper CB Bland G Pelch KE Gandhi SA BakenRa A . Effects of microplastic exposure on human digestive, reproductive, and respiratory health: A rapid systematic review. Environ Sci Technol. (2024) 58:22843–64. doi: 10.1021/acs.est.3c09524 39692326 PMC11697325

[20] Chen J Hendriks M Chatzis A Ramasamy SK Kusumbe AP . Bone vasculature and bone marrow vascular niches in health and disease. J Bone Mineral Res. (2020) 35:2103–20. doi: 10.1002/jbmr.4171 32845550

[21] Itkin T Gur-Cohen S Spencer JA Schajnovitz A Ramasamy SK Kusumbe AP . Distinct bone marrow blood vessels differentially regulate haematopoiesis. Nature. (2016) 532:323–8. doi: 10.1038/nature17624 27074509 PMC6450701

[22] Guo X Wang L Wang X Li D Wang H Xu H . Discovery and analysis of microplastics in human bone marrow. J Hazard Mater. (2024) 477:135266. doi: 10.1016/j.jhazmat.2024.135266 39079299

[23] Zhang W Liu K Zhou B Feng D Li Z Dai Z . RANKL/OPG axis as a therapeutic target for microplastic-induced bone loss: Mechanistic insights from transcriptomic and functional validation. Toxicol Lett. (2026) 415:111789. doi: 10.1016/j.toxlet.2025.111789 41412330

[24] Yang Q Peng Y Wu X Cao X Zhang P Liang Z . Microplastics in human skeletal tissues: Presence, distribution and health implications. Environ Int. (2025) 196:109316. doi: 10.1016/j.envint.2025.109316 39946929

[25] Pan C Hong R Wang K Shi Y Fan Z Liu T . Chronic exposure to polystyrene microplastics triggers osteoporosis by breaking the balance of osteoblast and osteoclast differentiation. Toxicology. (2025) 510:154017. doi: 10.1016/j.tox.2024.154017 39608439

[26] Giannandrea D Parolini M Citro V De Felice B Pezzotta A Abazari N . Nanoplastic impact on bone microenvironment: A snapshot from murine bone cells. J Hazard Mater. (2024) 462:132717. doi: 10.1016/j.jhazmat.2023.132717 37820528

[27] Zhang J Chu H Li R Liu C . Fine particulate matter and osteoporosis: Evidence, mechanisms, and emerging perspectives. Toxicological sciences: Off J Soc Toxicol. (2024) 202:157–66. doi: 10.1093/toxsci/kfae109 39222007

[28] Zhao S Kvale KF Zhu L Zettler ER Egger M Mincer TJ . The distribution of subsurface microplastics in the ocean. Nature. (2025) 641:51–61. doi: 10.1038/s41586-025-08818-1 40307520 PMC12043517

[29] Lamoree MH van Boxel J Nardella F Houthuijs KJ Brandsma SH Béen F . Health impacts of microplastic and nanoplastic exposure. Nat Med. (2025) 31:2873–87. doi: 10.1038/s41591-025-03902-5 40935856

[30] Chen D Wang P Liu S Wang R Wu Y Zhu A-X . Global patterns of lake microplastic pollution: Insights from regional human development levels. Sci Total Environ. (2024) 954:176620. doi: 10.1016/j.scitotenv.2024.176620 39362563

[31] Wang Z Zhang Y Kang S Yang L Shi H Tripathee L . Research progresses of microplastic pollution in freshwater systems. Sci Total Environ. (2021) 795:148888. doi: 10.1016/j.scitotenv.2021.148888 34328911

[32] Ten Hietbrink S Materić D Holzinger R Groeskamp S Niemann H . Nanoplastic concentrations across the North Atlantic. Nature. (2025) 643:412–6. doi: 10.1038/s41586-025-09218-1 40634739 PMC12240857

[33] Aransiola SA Victor-Ekwebelem MO Daza BX Oladoye PO Alli YA Bamisaye A . Micro- and nano-plastics pollution in the marine environment: Progresses, drawbacks and future guidelines. Chemosphere. (2025) 374:144211. doi: 10.1016/j.chemosphere.2025.144211 39977960

[34] Nawaz F Islam ZU Ghori SA Bahadur A Ullah H Ahmad M . Microplastic and nanoplastic pollution: Assessing translocation, impact, and mitigation strategies in marine ecosystems. Water Environ research: A Res Publ Water Environ Fed. (2025) 97:e70032. doi: 10.1002/wer.70032 39927485

[35] Okoffo ED Thomas KV . Quantitative analysis of nanoplastics in environmental and potable waters by pyrolysis-gas chromatography-mass spectrometry. J Hazard Mater. (2024) 464:133013. doi: 10.1016/j.jhazmat.2023.133013 37988869

[36] Vega-Herrera A Garcia-Torné M Borrell-Diaz X Abad E Llorca M Villanueva CM . Exposure to micro(nano)plastics polymers in water stored in single-use plastic bottles. Chemosphere. (2023) 343:140106. doi: 10.1016/j.chemosphere.2023.140106 37689148

[37] Jamison Hart MN Lenhart JJ . What’s in your water? A comparative analysis of micro- and nanoplastics in treated drinking water and bottled water. Sci Total Environ. (2026) 1011:181148. doi: 10.1016/j.scitotenv.2025.181148 41411791

[38] Bai C-L Wang D Luan Y-L Huang S-N Liu L-Y Guo Y . A review on micro- and nanoplastics in humans: Implication for their translocation of barriers and potential health effects. Chemosphere. (2024) 361:142424. doi: 10.1016/j.chemosphere.2024.142424 38795915

[39] Meng X Zheng X Mai W Gao J Fan Y Fu J . Micro- and nanoplastics differ in particle-mucus interactions: The sight on rheological properties, barrier dysfunction and microbiota dysbiosis. J Hazard Mater. (2025) 492:138130. doi: 10.1016/j.jhazmat.2025.138130 40220393

[40] Liu L Du R Niu L Li P Li Z-H . A latest review on micro- and nanoplastics in the aquatic environment: The comparative impact of size on environmental behavior and toxic effect. Bull Environ contamination Toxicol. (2024) 112:36. doi: 10.1007/s00128-024-03865-2 38353741

[41] Pelegrini K Pereira TCB Maraschin TG Teodoro LDS Basso NRDS De Galland GLB . Micro- and nanoplastic toxicity: A review on size, type, source, and test-organism implications. Sci Total Environ. (2023) 878:162954. doi: 10.1016/j.scitotenv.2023.162954 36948318

[42] Singh S Tiwari RR . Micro/nanoplastics and human health: A review of the evidence, consequences, and toxicity assessment. Food Chem toxicology: Int J published For Br Ind Biol Res Assoc. (2025) 203:115595. doi: 10.1016/j.fct.2025.115595 40505804

[43] Kögel T Bjorøy Ø Toto B Bienfait AM Sanden M . Micro- and nanoplastic toxicity on aquatic life: Determining factors. Sci Total Environ. (2020) 709:136050. doi: 10.1016/j.scitotenv.2019.136050 31887526

[44] Wieland S Ramsperger AFRM Gross W Lehmann M Witzmann T Caspari A . Nominally identical microplastic models differ greatly in their particle-cell interactions. Nat Commun. (2024) 15:922. doi: 10.1038/s41467-024-45281-4 38297000 PMC10830523

[45] Li X Qiu H Zhang P Song L Romero-Freire A He E . Role of heteroaggregation and internalization in the toxicity of differently sized and charged plastic nanoparticles to freshwater microalgae. Environ pollut (Barking Essex: 1987). (2023) 316:120517. doi: 10.1016/j.envpol.2022.120517 36309302

[46] Ramsperger AFRM Jasinski J Völkl M Witzmann T Meinhart M Jérôme V . Supposedly identical microplastic particles substantially differ in their material properties influencing particle-cell interactions and cellular responses. J Hazard Mater. (2022) 425:127961. doi: 10.1016/j.jhazmat.2021.127961 34986564

[47] Pérez-Ocampo J Tabares-Guevara JH Gómez-Gallego DM Taborda NA Hernandez JC . Understanding the nexus between microplastics and human health: A narrative review. CLEAN - Soil Air Water. (2026) 54:e70138. doi: 10.1002/clen.70138 41531421

[48] Saraceni A Schiavo V Mognetti B Cottone E Trianni A Beccari F . Microplastics interaction with bisphenol A: Adsorption, desorption, and *in vitro* biological effects. Sci Total Environ. (2025) 993:179971. doi: 10.1016/j.scitotenv.2025.179971 40609415

[49] Rocha CS Sanchez CA Souza MCO Treviso EM Savietto GH Devóz PP . Polystyrene nanoplastic co-exposed to BPA and BPS induces cytotoxicity, genotoxicity, and alters ROS production in HepG2 cells. Toxicol vitro: Int J published Assoc BIBRA. (2025) 109:106121. doi: 10.1016/j.tiv.2025.106121 40706907

[50] López-Vázquez J Rodil R Trujillo-Rodríguez MJ Quintana JB Cela R Miró M . Mimicking human ingestion of microplastics: Oral bioaccessibility tests of bisphenol A and phthalate esters under fed and fasted states. Sci Total Environ. (2022) 826:154027. doi: 10.1016/j.scitotenv.2022.154027 35217040

[51] DeLoid GM Yang Z Bazina L Kharaghani D Sadrieh F Demokritou P . Mechanisms of ingested polystyrene micro-nanoplastics (MNPs) uptake and translocation in an *in vitro* tri-culture small intestinal epithelium. J Hazard Mater. (2024) 473:134706. doi: 10.1016/j.jhazmat.2024.134706 38795489 PMC12036630

[52] Kharaghani D DeLoid GM He P Swenor B Bui TH Zuverza-Mena N . Toxicity and absorption of polystyrene micro-nanoplastics in healthy and Crohn’s disease human duodenum-chip models. J Hazard Mater. (2025) 490:137714. doi: 10.1016/j.jhazmat.2025.137714 40022921 PMC12051489

[53] Chen Y Williams AM Gordon EB Rudolph SE Longo BN Li G . Biological effects of polystyrene micro- and nano-plastics on human intestinal organoid-derived epithelial tissue models without and with M cells. Nanomedicine: Nanotechnology Biology Med. (2023) 50:102680. doi: 10.1016/j.nano.2023.102680 37105344 PMC10247512

[54] Paul MB Böhmert L Hsiao I-L Braeuning A Sieg H . Complex intestinal and hepatic *in vitro* barrier models reveal information on uptake and impact of micro-, submicro- and nanoplastics. Environ Int. (2023) 179:108172. doi: 10.1016/j.envint.2023.108172 37657408

[55] López de Las Hazas M-C Boughanem H Dávalos A . Untoward effects of micro- and nanoplastics: An expert review of their biological impact and epigenetic effects. Adv Nutr (Bethesda Md). (2022) 13:1310–23. doi: 10.1093/advances/nmab154 34928307 PMC9340974

[56] Hu L Feng X Lan Y Zhang J Nie P Xu H . Co-exposure with cadmium elevates the toxicity of microplastics: Trojan horse effect from the perspective of intestinal barrier. J Hazard Mater. (2024) 466:133587. doi: 10.1016/j.jhazmat.2024.133587 38280329

[57] Qiu W Ye J Su Y Zhang X Pang X Liao J . Co-exposure to environmentally relevant concentrations of cadmium and polystyrene nanoplastics induced oxidative stress, ferroptosis and excessive mitophagy in mice kidney. Environ pollut (Barking Essex: 1987). (2023) 333:121947. doi: 10.1016/j.envpol.2023.121947 37270049

[58] Qiao J Chen R Wang M Bai R Cui X Liu Y . Perturbation of gut microbiota plays an important role in micro/nanoplastics-induced gut barrier dysfunction. Nanoscale. (2021) 13:8806–16. doi: 10.1039/d1nr00038a 33904557

[59] Hsu W-H Chen Y-Z Chiang Y-T Chang Y-T Wang Y-W Hsu K-T . Polystyrene nanoplastics disrupt the intestinal microenvironment by altering bacteria-host interactions through extracellular vesicle-delivered microRNAs. Nat Commun. (2025) 16:5026. doi: 10.1038/s41467-025-59884-y 40494850 PMC12152142

[60] Kim DH Lee S Ahn J Kim JH Lee E Lee I . Transcriptomic and metabolomic analysis unveils nanoplastic-induced gut barrier dysfunction via STAT1/6 and ERK pathways. Environ Res. (2024) 249:118437. doi: 10.1016/j.envres.2024.118437 38346486

[61] Cui M He Q Wang Z Yu Y Gao H Liu Z . Mucin2 regulated by Ho1/p38/IL-10 axis plays a protective role in polystyrene nanoplastics-mediated intestinal toxicity. Environ pollut (Barking Essex: 1987). (2023) 330:121808. doi: 10.1016/j.envpol.2023.121808 37182580

[62] Chen Y Xuan Y Chen X Wu M Xu J Chen J . Gastrointestinal digestion potentiates nanoplastic-induced intestinal barrier dysfunction and macrophage-driven inflammation. J Hazard Mater. (2026) 503:141129. doi: 10.1016/j.jhazmat.2026.141129 41544594

[63] Du L Liu H Song X Feng X Xu H Tang W . Developments in the field of intestinal toxicity and signaling pathways associated with rodent exposure to micro(nano)plastics. Toxicology. (2024) 507:153883. doi: 10.1016/j.tox.2024.153883 38996996

[64] Huang Z Weng Y Shen Q Zhao Y Jin Y . Microplastic: A potential threat to human and animal health by interfering with the intestinal barrier function and changing the intestinal microenvironment. Sci Total Environ. (2021) 785:147365. doi: 10.1016/j.scitotenv.2021.147365 33933760

[65] Varma S Duttaroy AK Basak S . Human exposure to micro- and nanoplastics: a mechanistic perspective of health risks associated with metabolic and reproductive functions. Sci Total Environ. (2025) 989:179879. doi: 10.1016/j.scitotenv.2025.179879 40505506

[66] Jing J Zhang L Han L Wang J Zhang W Liu Z . Polystyrene micro-/nanoplastics induced hematopoietic damages via the crosstalk of gut microbiota, metabolites, and cytokines. Environ Int. (2022) 161:107131. doi: 10.1016/j.envint.2022.107131 35149446

[67] Sokolova V Loza K Knuschke T Heinen-Weiler J Jastrow H Hasenberg M . A systematic electron microscopic study on the uptake of barium sulphate nano-, submicro-, microparticles by bone marrow-derived phagocytosing cells. Acta Biomater. (2018) 80:352–63. doi: 10.1016/j.actbio.2018.09.026 30240952

[68] Martin JD Toh K Martin MR Chen P Wang C Igarashi K . Bone marrow vessels are hyperpermeable to macromolecules and nanoscale medicine in a size-dependent manner. J Controlled Release. (2025) 382:113669. doi: 10.1016/j.jconrel.2025.113669 40158811

[69] Chen D Liu Y Zhang Z Liu Z Fang X He S . NIR-II fluorescence imaging reveals bone marrow retention of small polymer nanoparticles. Nano Lett. (2021) 21:798–805. doi: 10.1021/acs.nanolett.0c04543 33346668

[70] Cao J Yang Q Jiang J Dalu T Kadushkin A Singh J . Coronas of micro/nano plastics: a key determinant in their risk assessments. Part Fibre Toxicol. (2022) 19:55. doi: 10.1186/s12989-022-00492-9 35933442 PMC9356472

[71] Brouwer H Porbahaie M Boeren S Busch M Bouwmeester H . The *in vitro* gastrointestinal digestion-associated protein corona of polystyrene nano- and microplastics increases their uptake by human THP-1-derived macrophages. Part Fibre Toxicol. (2024) 21:4. doi: 10.1186/s12989-024-00563-z 38311718 PMC10838446

[72] Francia V Yang K Deville S Reker-Smit C Nelissen I Salvati A . Corona composition can affect the mechanisms cells use to internalize nanoparticles. ACS Nano. (2019) 13:11107–21. doi: 10.1021/acsnano.9b03824 31525954 PMC6812477

[73] Sterin EH Weinstein LA Tiwari A Kramarenko GC Chowdhury CR Li K . Influence of the protein corona on hematopoietic stem and progenitor cell uptake and macrophage clearance of membrane-wrapped nanoparticles. PNAS. (2025) 122:e2507922122. doi: 10.1073/pnas.2507922122 41021803 PMC12519180

[74] Guo X Cheng C Chen L Cao C Li D Fan R . Metabolomic characteristics in human CD34(+) hematopoietic stem/progenitor cells exposed to polystyrene nanoplastics. Food Chem Toxicology: Int J Published For Br Ind Biol Res Assoc. (2023) 177:113817. doi: 10.1016/j.fct.2023.113817 37164248

[75] Christodoulou MC Stylianou M Voukkali I Naddeo V Barceló D Kepertis C . Unveiling the presence of micro and nanoplastics in human biological matrices: A systematic review covering the latest five years from 2020 to 2025. Sci Total Environ. (2026) 1013:181304. doi: 10.1016/j.scitotenv.2025.181304 41468853

[76] Huang F Wong P Li J Lv Z Xu L Zhu G . Osteoimmunology: The correlation between osteoclasts and the Th17/Treg balance in osteoporosis. J Cell Mol Med. (2022) 26:3591–7. doi: 10.1111/jcmm.17399 35633138 PMC9258696

[77] Wei Y Wu Y Zhu D Zhang J Li X Wang M . Polystyrene-nanoplastics-induced unfolded protein response in monocyte-derived macrophages mediates pulmonary fibrosis via oxidative-stress-dependent IL-6 secretion. J Hazard Mater. (2026) 506:141476. doi: 10.1016/j.jhazmat.2026.141476 41747702

[78] Liu L Liu B Zhang B Ye Y Jiang W . Polystyrene micro(nano)plastics damage the organelles of RBL-2H3 cells and promote MOAP-1 to induce apoptosis. J Hazard Mater. (2022) 438:129550. doi: 10.1016/j.jhazmat.2022.129550 35999725

[79] Guo X Cheng C Wang L Li D Fan R Wei X . Polystyrene nanoplastics induce haematotoxicity with cell oxeiptosis and senescence involved in C57BL/6J mice. Environ Toxicol. (2023) 38:2487–98. doi: 10.1002/tox.23886 37466197

[80] Li L Su Y Huang J Xu W . Polystyrene microplastics induces senescence of osteocytes by activating the cyclooxygenase-2 signaling pathway. Ecotoxicology Environ Saf. (2025) 300:118466. doi: 10.1016/j.ecoenv.2025.118466 40466333

[81] Rehman MFU Khan MM Khan MM . Impact of microplastics and nanoplastics on human health: Mechanistic insights and exposure pathways. Toxicol Lett. (2025) 414:111769. doi: 10.1016/j.toxlet.2025.111769 41203090

[82] Xu J Ze X Zhao L Sheng L Ze Y . Titanium dioxide nanoparticles oral exposure induce osteoblast apoptosis, inhibit osteogenic ability and increase lipogenesis in mouse. Ecotoxicology Environ Saf. (2024) 277:116367. doi: 10.1016/j.ecoenv.2024.116367 38669870

[83] Banerjee A Shelver WL . Micro- and nanoplastic induced cellular toxicity in mammals: A review. Sci Total Environ. (2021) 755:142518. doi: 10.1016/j.scitotenv.2020.142518 33065507

[84] Ninomiya H Fukuda S Nishida-Fukuda H Shibata Y Sato T Nakamichi Y . Osteoprotegerin secretion and its inhibition by RANKL in osteoblastic cells visualized using bioluminescence imaging. Bone. (2025) 191:117319. doi: 10.1016/j.bone.2024.117319 39500402

[85] Wang X Sun B Wang Y Gao P Song J Chang W . Research progress of targeted therapy regulating Th17/Treg balance in bone immune diseases. Front Immunol. (2024) 15:1333993. doi: 10.3389/fimmu.2024.1333993 38352872 PMC10861655

[86] Wolff CM Singer D Schmidt A Bekeschus S . Immune and inflammatory responses of human macrophages, dendritic cells, and T-cells in presence of micro- and nanoplastic of different types and sizes. J Hazard Mater. (2023) 459:132194. doi: 10.1016/j.jhazmat.2023.132194 37572607

[87] Qoreishi M Panahi M Dorodi O Ghanbari N Jousheghan SS . Involvement of NF-κB/NLRP3 axis in the progression of aseptic loosening of total joint arthroplasties: a review of molecular mechanisms. Naunyn-Schmiedeberg’s Arch Pharmacol. (2022) 395:757–67. doi: 10.1007/s00210-022-02232-4 35377011

[88] Yang Q Dai H Cheng Y Wang B Xu J Zhang Y . Oral feeding of nanoplastics affects brain function of mice by inducing macrophage IL-1 signal in the intestine. Cell Rep. (2023) 42:112346. doi: 10.1016/j.celrep.2023.112346 37022934

[89] Yang Q Dai H Wang B Xu J Zhang Y Chen Y . Nanoplastics shape adaptive anticancer immunity in the colon in mice. Nano Lett. (2023) 23:3516–23. doi: 10.1021/acs.nanolett.3c00644 37043775

[90] Mori G D’Amelio P Faccio R Brunetti G . The interplay between the bone and the immune system. Clin Dev Immunol. (2013) 2013:720504. doi: 10.1155/2013/720504 23935650 PMC3725924

[91] Miossec P Korn T Kuchroo VK . Interleukin-17 and type 17 helper T cells. N Engl J Med. (2009) 361:888–98. doi: 10.1056/NEJMra0707449 19710487

[92] Wang Z Wei Y Lei L Zhong J Shen Y Tan J . RANKL expression of primary osteoblasts is enhanced by an IL-17-mediated JAK2/STAT3 pathway through autophagy suppression. Connective Tissue Res. (2021) 62:411–26. doi: 10.1080/03008207.2020.1759562 32370570

[93] Liu Y Ouyang Y You W Liu W Cheng Y Mai X . Physiological roles of human interleukin-17 family. Exp Dermatol. (2024) 33:e14964. doi: 10.1111/exd.14964 37905720

[94] Maharana T Taranath A Fernandes CSE Mishra P Muralidaran Y . Micro- and nanoplastic-induced mitochondrial dysfunction and organelle miscommunication: A toxicological perspective. Toxicology. (2026) 519:154306. doi: 10.1016/j.tox.2025.154306 41086899

[95] K C PB Maharjan A Acharya M Lee D Kusma S Gautam R . Polytetrafluorethylene microplastic particles mediated oxidative stress, inflammation, and intracellular signaling pathway alteration in human derived cell lines. Sci Total Environ. (2023) 897:165295. doi: 10.1016/j.scitotenv.2023.165295 37419366

[96] Wang X Ren X-M He H Li F Liu K Zhao F . Cytotoxicity and pro-inflammatory effect of polystyrene nano-plastic and micro-plastic on RAW264.7 cells. Toxicology. (2023) 484:153391. doi: 10.1016/j.tox.2022.153391 36503103

[97] Ormsby RT Solomon LB Yang D Crotti TN Haynes DR Findlay DM . Osteocytes respond to particles of clinically-relevant conventional and cross-linked polyethylene and metal alloys by up-regulation of resorptive and inflammatory pathways. Acta Biomater. (2019) 87:296–306. doi: 10.1016/j.actbio.2019.01.047 30690207

[98] Liu H Wang D Li J Wang Y Liang Z Chen X . Gasdermin D-dependent macrophage pyroptosis mediates polystyrene microplastics-induced pulmonary fibrosis. Int Immunopharmacol. (2026) 172:116189. doi: 10.1016/j.intimp.2026.116189 41539003

[99] Alijagic A Hedbrant A Persson A Larsson M Engwall M Särndahl E . NLRP3 inflammasome as a sensor of micro- and nanoplastics immunotoxicity. Front Immunol. (2023) 14:1178434. doi: 10.3389/fimmu.2023.1178434 37143682 PMC10151538

[100] Busch M Bredeck G Waag F Rahimi K Ramachandran H Bessel T . Assessing the NLRP3 inflammasome activating potential of a large panel of micro- and nanoplastics in THP-1 cells. Biomolecules. (2022) 12(8):1095. doi: 10.3390/biom12081095 36008988 PMC9406042

[101] Adler MY Issoual I Rückert M Deloch L Meier C Tschernig T . Effect of micro- and nanoplastic particles on human macrophages. J Hazard Mater. (2024) 471:134253. doi: 10.1016/j.jhazmat.2024.134253 38642497

[102] Ma Q Lim CS . Molecular activation of NLRP3 inflammasome by particles and crystals: a continuing challenge of immunology and toxicology. Annu Rev Pharmacol Toxicol. (2024) 64:417–33. doi: 10.1146/annurev-pharmtox-031023-125300 37708431 PMC10842595

[103] Chen Q Liu Y Bi L Jin L Peng R . Understanding the mechanistic roles of microplastics combined with heavy metals in regulating ferroptosis: Adding new paradigms regarding the links with diseases. Environ Res. (2024) 242:117732. doi: 10.1016/j.envres.2023.117732 37996004

[104] Wang L Zhang X Xu M Zheng G Chen J Li S . Implication of ferroptosis in hepatic toxicity upon single or combined exposure to polystyrene microplastics and cadmium. Environ pollut (Barking Essex: 1987). (2023) 334:122250. doi: 10.1016/j.envpol.2023.122250 37487871

[105] Yang H Niu S Guo M Liu C Xue Y . Toxic mechanisms of nanoparticle-induced ferroptosis and current research challenges: a critical review. Environ pollut (Barking Essex: 1987). (2025) 387:127328. doi: 10.1016/j.envpol.2025.127328 41161466

[106] Kim B Park K-M Lee H Hyun Y-M . Microplastic-induced macrophage dysfunction drives lung tumor progression through glutathione imbalance. ACS Nano. (2026) 20:5489–505. doi: 10.1021/acsnano.5c15425 41670235 PMC12947730

[107] Pelepenko LE de Oliveira MC Masaro DA Lustosa GMMM Mazon T Castilho RF . Effects of microplastics on the bones: a comprehensive review. Osteoporosis International: A J Established as Result Cooperation Between Eur Foundation For Osteoporosis Natl Osteoporosis Foundation USA. (2025) 36:1327–45. doi: 10.1007/s00198-025-07580-4 40553183

[108] Zhang Z Xu M Wang L Gu W Li X Han Z . Continuous oral exposure to micro- and nanoplastics induced gut microbiota dysbiosis, intestinal barrier and immune dysfunction in adult mice. Environ Int. (2023) 182:108353. doi: 10.1016/j.envint.2023.108353 38035535

[109] Yan Z Liu R Zhang Z-A Dai T Zhu D Zhang Y . Intestinal microplastic retention reshapes gut microbial ecology through surface-associated colonization and additive leaching. Environ Sci Technol. (2026) 60:3974–86. doi: 10.1021/acs.est.5c16988 41617658

[110] Chen J Cheng Y Fu R Chen X Zhang P Lu Y . Multiomics reveals nonphagocytosable microplastics induce colon inflammatory injury via bile acid-gut microbiota interactions and barrier dysfunction. ACS Appl Materials Interfaces. (2025) 17:44138–59. doi: 10.1021/acsami.5c07250 40682529 PMC12332821

[111] Xie X Chen X Wang Z Chen Y Li J . The role of gut microbiota-immune-endocrine crosstalk in the pathogenesis of osteoporosis. Front Immunol. (2026) 17:1813653. doi: 10.3389/fimmu.2026.1813653 42099587 PMC13143884

[112] Bott KN Feldman E de Souza RJ Comelli EM Klentrou P Peters SJ . Lipopolysaccharide-induced bone loss in rodent models: a systematic review and meta-analysis. J Bone Mineral Res. (2023) 38:198–213. doi: 10.1002/jbmr.4740 36401814 PMC10107812

[113] Kondo T Chiba T Tousen Y . Short-chain fatty acids, acetate and propionate, directly upregulate osteoblastic differentiation. Int J Food Sci Nutr. (2022) 73:800–8. doi: 10.1080/09637486.2022.2078285 35616294

[114] Lucas S Omata Y Hofmann J Böttcher M Iljazovic A Sarter K . Short-chain fatty acids regulate systemic bone mass and protect from pathological bone loss. Nat Commun. (2018) 9:55. doi: 10.1038/s41467-017-02490-4 29302038 PMC5754356

[115] Montalvany-Antonucci CC Duffles LF de Arruda JAA Zicker MC de Oliveira S Macari S . Short-chain fatty acids and FFAR2 as suppressors of bone resorption. Bone. (2019) 125:112–21. doi: 10.1016/j.bone.2019.05.016 31100533

[116] Ticinesi A Siniscalchi C Meschi T Nouvenne A . Gut microbiome and bone health: update on mechanisms, clinical correlations, and possible treatment strategies. Osteoporosis International: A J Established as Result Cooperation Between Eur Foundation For Osteoporosis Natl Osteoporosis Foundation USA. (2025) 36:167–91. doi: 10.1007/s00198-024-07320-0 39643654

[117] Ling C-W Miao Z Xiao M-L Zhou H Jiang Z Fu Y . The association of gut microbiota with osteoporosis is mediated by amino acid metabolism: Multiomics in a large cohort. J Clin Endocrinol Metab. (2021) 106:e3852–64. doi: 10.1210/clinem/dgab492 34214160

[118] Ullah S Ahmad S Guo X Ullah S Ullah S Nabi G . A review of the endocrine disrupting effects of micro and nano plastic and their associated chemicals in mammals. Front Endocrinol. (2022) 13:1084236. doi: 10.3389/fendo.2022.1084236 36726457 PMC9885170

[119] Wang Q Kong P Wang Q Ruan J Yang W Teng X . Polyethylene microplastic exposure disrupts sex and gut hormones via gut microbial and metabolic pathways. FASEB J. (2026) 40:e71632. doi: 10.1096/fj.202502063R 41801048

[120] Shahsavari S Akbari-Adergani B Shafaroodi H Akbari N Basaran B Sadighara M . A systematic review on the effect of microplastics on the hypothalamus-pituitary-ovary axis based on animal studies. Toxicol Lett. (2025) 413:111745. doi: 10.1016/j.toxlet.2025.111745 41101573

[121] Wang W Ma A Zhai C Lan W Liu Z Yang Z . Bisphenol A and di-n-butyl phthalate disrupt bone homeostasis bidirectionally via CD36-mediated BMSCs autophagy inhibition and exosome-promoted osteoclastogenesis. J Hazard Mater. (2025) 497:139703. doi: 10.1016/j.jhazmat.2025.139703 40902528

[122] Zhang Y Zheng L Cheng D Lei C Li H Zhou J . Chronic di(2-ethylhexyl) phthalate exposure at environmental-relevant doses induces osteoporosis by disturbing the differentiation of bone marrow mesenchymal stem cells. Sci Total Environ. (2024) 914:169918. doi: 10.1016/j.scitotenv.2024.169918 38190899

[123] Zhang J Liu L Dai X Li B Zhang S Yu Y . Thyroid and parathyroid function disorders induced by short-term exposure of microplastics and nanoplastics: Exploration of toxic mechanisms and early warning biomarkers. J Hazard Mater. (2024) 476:134960. doi: 10.1016/j.jhazmat.2024.134960 38901250

[124] Fu F Chen Y Luo H Ruan H . Micro/nanoplastics induce thyroid follicular cell pyroptosis to trigger thyrotoxicity by activating NF-κB signaling. Ann Med. (2026) 58:2624175. doi: 10.1080/07853890.2026.2624175 41700422 PMC12915396

[125] Li B Lyu P Tang J Li J Ouchi T Fan Y . The potential role and therapeutic relevance of cellular senescence in skeletal pathophysiology. Journals Gerontology Ser A Biol Sci Med Sci. (2024) 79(4):glae037. doi: 10.1093/gerona/glae037 38306619

[126] Chandra A Law SF Pignolo RJ . Changing landscape of hematopoietic and mesenchymal cells and their interactions during aging and in age-related skeletal pathologies. Mech Ageing Dev. (2025) 225:112059. doi: 10.1016/j.mad.2025.112059 40220914 PMC12103995

[127] Upadhyay P Suhail A Khanal P Kumar S . Mechanistic insights and biomarker discovery in immune cell aging and age-associated diseases. Front Immunol. (2025) 16:1637191. doi: 10.3389/fimmu.2025.1637191 41132677 PMC12540116

[128] Rao X Cai X . Aging-driven inter-organ crosstalk in postmenopausal osteoporosis: From immunometabolic drift to multisystem frailty. FASEB J. (2026) 40:e71541. doi: 10.1096/fj.202505069R 41721711 PMC12924573

[129] Li D Zheng X Lin D Cheng Y Wang Z Chen Y . Immune reprogramming in the bone marrow microenvironment: a new perspective on the bone immune microenvironment of postmenopausal osteoporosis. Front Immunol. (2026) 17:1766460. doi: 10.3389/fimmu.2026.1766460 41836370 PMC12982044

[130] da Silva Brito WA Mutter F Wende K Cecchini AL Schmidt A Bekeschus S . Consequences of nano and microplastic exposure in rodent models: the known and unknown. Part Fibre Toxicol. (2022) 19:28. doi: 10.1186/s12989-022-00473-y 35449034 PMC9027452

[131] Yadav H Sethulekshmi S Shriwastav A . Estimation of microplastic exposure via the composite sampling of drinking water, respirable air, and cooked food from Mumbai, India. Environ Res. (2022) 214:113735. doi: 10.1016/j.envres.2022.113735 35753373

[132] Danopoulos E Twiddy M West R Rotchell JM . A rapid review and meta-regression analyses of the toxicological impacts of microplastic exposure in human cells. J Hazard Mater. (2022) 427:127861. doi: 10.1016/j.jhazmat.2021.127861 34863566

[133] Djouina M Pichavant M Waxin C Dehaut A Loison S Driencourt E . Ingestion of a human-relevant mixture of environmentally sourced microplastics promotes inflammation and tumorigenesis in the mouse colon. Environ pollut (Barking Essex: 1987). (2026) 395:127794. doi: 10.1016/j.envpol.2026.127794 41672396

[134] Deng Y Chen H Huang Y Zhang Y Ren H Fang M . Long-term exposure to environmentally relevant doses of large polystyrene microplastics disturbs lipid homeostasis via bowel function interference. Environ Sci Technol. (2022) 56:15805–17. doi: 10.1021/acs.est.1c07933 36282942

[135] Zhang G Sun J Zhan Y Xiao B Liu H Wang L . Bioaccumulation, trophic transfer, and health risk assessment of microplastics in the food web of Wuliangsuhai Lake, China: Higher risk for children. Environ pollut (Barking Essex: 1987). (2026) 395:127787. doi: 10.1016/j.envpol.2026.127787 41662944

[136] Zhang Q Lang Y Tang X Cheng W Cheng Z Rizwan M . Polystyrene microplastic-induced endoplasmic reticulum stress contributes to growth plate endochondral ossification disorder in young rat. Environ Toxicol. (2024) 39:3314–29. doi: 10.1002/tox.24182 38440912

[137] Li Y Piao G Hu F Chen W Wang Q Zhang X . The silent invasion of microplastics polyvinyl chloride and polyethylene terephthalate: Potential impact on osteoporosis. J Hazard Mater. (2025) 492:138074. doi: 10.1016/j.jhazmat.2025.138074 40158506

[138] Mohamed Nor NH Kooi M Diepens NJ Koelmans AA . Lifetime accumulation of microplastic in children and adults. Environ Sci Technol. (2021) 55:5084–96. doi: 10.1021/acs.est.0c07384 33724830 PMC8154366

[139] Bi S Liu S Liu E Xiong J Xu Y Wu R . Adsorption behavior and mechanism of heavy metals onto microplastics: A meta-analysis assisted by machine learning. Environ pollut (Barking Essex: 1987). (2024) 360:124634. doi: 10.1016/j.envpol.2024.124634 39084591

[140] Muthuraja R Ou B Thangavelu M Narhayanan TN Chittamart N Janjaroen D . Effects of particle size and aging on heavy metal adsorption by polypropylene and polystyrene microplastics under varying environmental conditions. Chemosphere. (2024) 369:143843. doi: 10.1016/j.chemosphere.2024.143843 39617328

[141] Li X Hu S Yu Z He F Zhao X Liu R . New evidence for the mechanisms of nanoplastics amplifying cadmium cytotoxicity: Trojan horse effect, inflammatory response, and calcium imbalance. Environ Sci Technol. (2025) 59:9471–85. doi: 10.1021/acs.est.5c01254 40350783

[142] Chen G Fu Q Tan X Yang H Luo Y Shen M . Speciation and release risk of heavy metals bonded on simulated naturally-aged microplastics prepared from artificially broken macroplastics. Environ pollut (Barking Essex: 1987). (2022) 295:118695. doi: 10.1016/j.envpol.2021.118695 34921945

[143] Wang W-J Wu C-C Jung W-T Lin C-Y . The associations among lead exposure, bone mineral density, and FRAX score: NHANES, 2013 to 2014. Bone. (2019) 128:115045. doi: 10.1016/j.bone.2019.115045 31446117

[144] Cui H Jiang X Cao J Yang W Yang B Li M . Comparative analysis of metabolic dysfunctions associated with pristine and aged polyethylene microplastic exposure via the liver-gut axis in mice. ACS Nano. (2025) 19:14272–83. doi: 10.1021/acsnano.5c00945 40189833

[145] Senathirajah K Attwood S Bhagwat G Carbery M Wilson S Palanisami T . Estimation of the mass of microplastics ingested - a pivotal first step towards human health risk assessment. J Hazard Mater. (2021) 404:124004. doi: 10.1016/j.jhazmat.2020.124004 33130380

